# Human Physiology

**Published:** 1850-12

**Authors:** 


Art. VI.—*Human Physiology.* By Robley Dunglison, M. D., Professor of the Institutes of Medicine in Jefferson Medical College, Philadelphia. With nearly five hundred illustrations. Seventh edition, thoroughly revised, and extensively modified and enlarged. In two volumes.

We have expressed a favorable opinion of this work, in noticing some of the earlier editions. We hold the laborious zeal of Professor Dunglison for the advancement of the medical profession in high estimation, and feel that he deserves the respect and esteem of his medical brethren throughout the Union. He has but little faculty for originating discoveries, but he possesses great powers for gathering and concentrating all the lights of medical science as they spring to view from various quarters.—These qualities have deservedly given him a high position in American medical literature, and the repeated calls for new editions of his various works are substantial evidences of his extensive popularity. We have before us the seventh edition of the work on human physiology, and are gratified in being able to say that it is posted up pretty well to the present position of the science. The explorations and investigations of inquirers in physiology, are at this time beyond all former example, and the science is progressing at a rapid and, we hope, a truthful rate. It is, of all others, the most fascinating and delightful science, and it is no matter of amazement to us, that so many able men, in all countries, are devoting all their powers to its illustration, and its truths. Bérard, Paget, Bernard, Bischoff, Agassiz, and a host of others, have greatly extended our knowledge of the truths of physiology; they have revealed new light, cleared up many obscurities, and corrected a vast number of errors. It has been the object of Professor Dunglison, in preparing this seventh edition of his work, to post up the condition of physiology to the present date, and he has succeeded very well in this design.

We are prepared to endorse the following remarks in the preface to the seventh edition: "On no previous revision of this work has the author bestowed more care than on the present. In the successive editions, it was of course, necessary to incorporate the different facts and principles, which had been added from time to time, to the science; and this rendered it difficult to preserve throughout the evenness of style, which is so desirable in every treatise, and more especially in one that is placed in the hands of so many of the younger portion of scientific inquirers.

"Perhaps at no time in the history of the science, have observers been more numerous, energetic, and discriminating than in the last few years. Many modifications of fact and inference have consequently taken place, which it has been necessary for the author to record, and to express his views in relation thereto. Especially has he endeavored to note the phenomena that have presented themselves to the most accurate observers, and to deduce from them laws which may tend to enlarge the boundaries of the science; he has not, however, felt himself at liberty to discard the results of the observations of all former anthropologists, or the opinions they had embraced in regard to the various functions. It not unfrequently, indeed, happens, that in ignorance of the history of the science, views are esteemed new, which had been promulgated by earlier investigators. He has, therefore, in an encyclopedic work like the present, retained many of those opinions, whilst he has labored to do especial justice to such as have emanated from more recent inquirers."

We thought it was due to professor Dunglison, to let him thus express the character of his work, and in the remarks we have quoted, he does so in appropriate terms. We consider this edition a valuable contribution to our stock of physiological works, and while thanking the author for his labors, we cordially commend his book to medical readers.

The publishers have illustrated this edition in their usual style, but we hope the time is not very distant, when a higher state of art will be found necessary in illustrating medical books. Much remains to be done in this department.

